# Metastatic Adenocarcinoma of Mandible with Unknown Primary Origin (CUP Syndrome): a Rare Case Report

**DOI:** 10.30476/dentjods.2023.97749.2034

**Published:** 2023-12-01

**Authors:** Saede Atarbashi-Moghadam, Mohammad Jafarian, Shaghayegh Dowdani

**Affiliations:** 1 Dept. of Oral and Maxillofacial Pathology, School of Dentistry, Shahid Beheshti University of Medical Sciences, Tehran, Iran; 2 Dept. of Oral and Maxillofacial Surgery, Taleghani Medical Center, School of Dentistry, Shahid Beheshti University of Medical Sciences, Tehran, Iran; 3 Graduated Student, School of Dentistry, Shahid Beheshti University of Medical Sciences, Tehran, Iran

**Keywords:** Oral cancer, Adenocarcinoma, Metastasis, Mandible

## Abstract

Metastatic lesions of the jaws are a diagnostic challenge because of their scarcity and uncharacteristic clinical-radiographic features. Carcinoma of unknown primary origin (CUP) is characterized by the existence of metastatic disease with no recognized primary neoplasm after a comprehensive work-up. CUP shows a poor prognosis with limited treatment choices. This paper presents a 64-year-old male with a chief complaint of paresthesia of the chin and lower lip. Panoramic radiography showed an ill-defined radiolucency in the left mandibular molar area and the residue of the first molar root. Microscopic examination demonstrated features of mucin-producing adenocarcinoma and was not similar to common neoplasms of the jaw. The whole-body scan revealed multiple osseous uptakes. CDX2 was diffusely positive. However, in the end, the origin of the primary tumor was not determined. Considering the aforementioned data, the diagnosis of metastatic adenocarcinoma with unknown primary origin was made. CUP of the oral cavity is an extremely rare event. The possibility of metastasis should be raised in a patient who complains of paresthesia. Awareness of the clinical and histopathologic features of these malignancies is crucial for clinicians and pathologists to have a proper diagnosis.

## Introduction

Jaw metastasis has great importance because it may be the only sign of an undiagnosed primary malignancy. Clinical signs and symptoms are variable, and the lesion may be asymptomatic. The most common radiographic feature is an ill-defined radiolucency [ 1 ]. Metastatic lesions of the jaws show posterior mandibular predilection. They are more common in men and the elderly [ 2 ]. Factors other than the frequency of neoplasms may be present in oral metastasis, such as biological behavior of cancer, aggressiveness, and a tendency for particular regions of oral cavity [ 3 - 4 ]. CUP is a diverse group of malignancies characterized by the existence of metastatic disease with no recognized primary neoplasm at initial presentation. It comprises 2–3% of all epithelial cancers [ 5 - 6 ]. CUP shows a poor prognosis with limited treatment choices [ 6 ]. Dental practitioners have a vital role in the diagnosis of cancer patients, mainly those with oral metastasis and silent primary malignancies [ 2 ]. This paper presents a case of metastatic adenocarcinoma with unknown primary origin of the left molar region of the mandible affecting a 64-year-old man.

## Case Presentation

**Figure 1:**
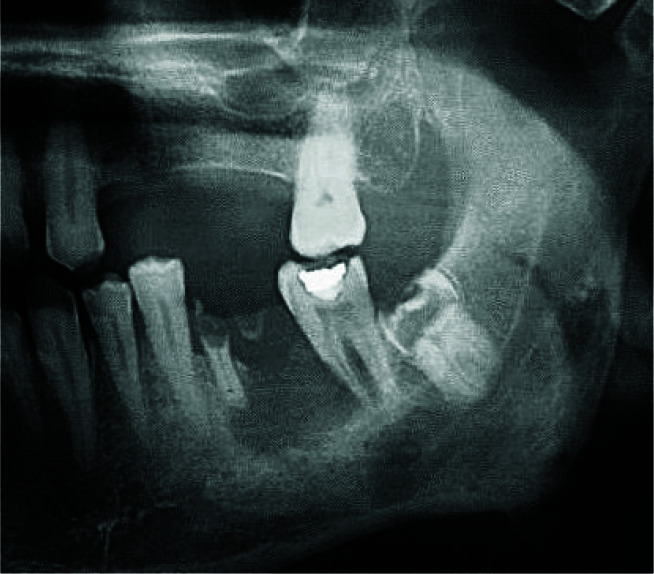
Panoramic radiograph shows an ill-defined radiolucent lesion in area of #18, #19 with bone sclerosis, residue of first molar root and resorption of mesial root of #18

**Figure 2:**
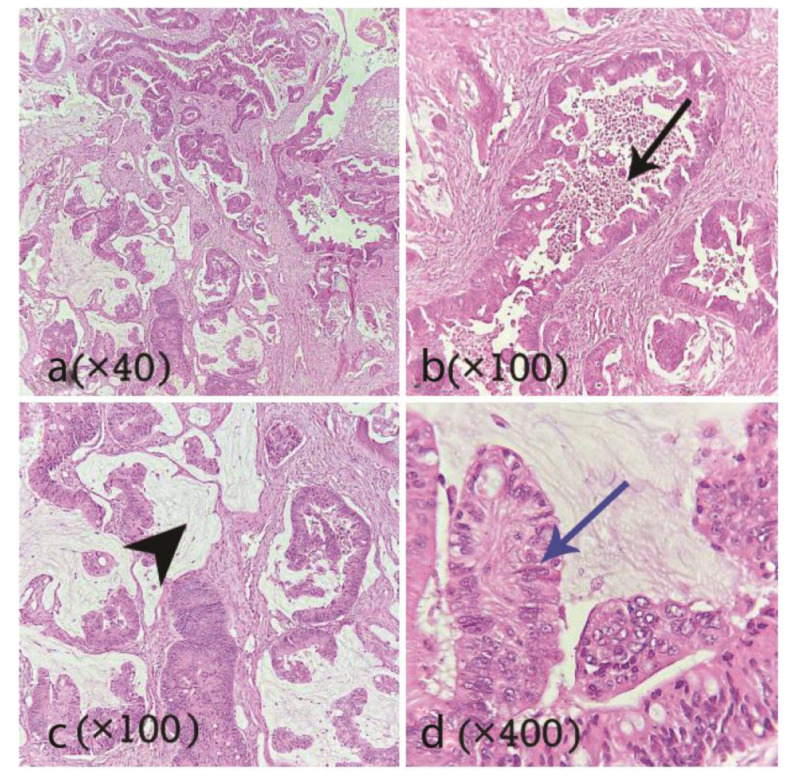
**a, b:** Histopathologic sections demonstrate ductal structures (black arrow) lined by pseudo-stratified columnar epithelium, **c:** Mucoid stroma (arrowhead), **d:** Tall columnar cells with vesicular nuclei (blue arrow)

**Figure 3:**
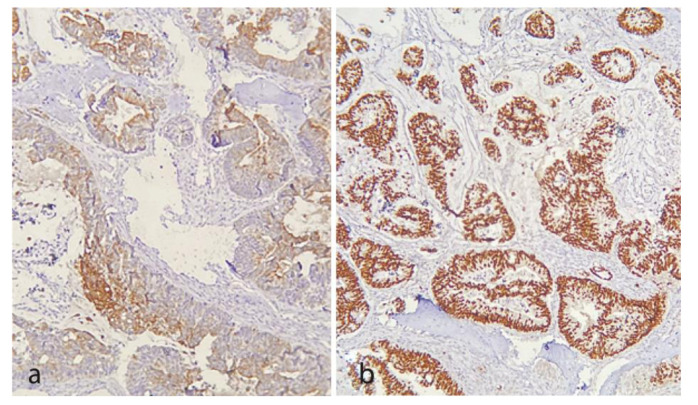
**a:** CK20 showed a patchy reaction (100×), **b:** CDX2 was diffusely and strongly positive (100×)

A 64-year-old man with a chief complaint of paresthesia of the chin and lower lip area was referred to a private dental clinic (Tehran, Iran) in May 2018. He had a history of Wegener's granulomatosis and was taking methotrexate, prednisone, and Calcium D. Extra-oral examination was normal and the intraoral examination revealed mild buccal expansion without any mucosal erosion or ulcer. There was no cervical lymphadenopathy. Panoramic radiography revealed a radiolucent lesion with ill-defined borders in areas #18 and #19 with bone sclerosis and residue of the first molar root. Resorption of the mesial root of the second left molar is also evident (Figure 1). Due to numb chin syndrome and radiographic features, a provisional diagnosis of the inflammatory periapical lesion, lymphoma, aggressive central giant cell granuloma, and odontogenic carcinoma were made, and an incisional biopsy was performed under local anesthesia. Histopathologic examination showed a malignant epithelial neoplasm composed of cribriform and ductal architectures lined by pseudo-stratified columnar epithelium with vesicular nuclei. Tumoral giant cells and goblet cells existed. The stroma was fibrous to mucoid and contained many foamy histiocytes. Tumoral islands were admixed with bony trabeculae. Hemorrhage, chronic inflammatory cell infiltration, and cholesterol clefts were also seen (Figure 2). Based on microscopic features and the intraosseous nature of the lesion, an overall diagnosis of adenocarcinoma was rendered. A whole-body scan and immunohistochemical (IHC) staining for CK7, CK20, Napsin A, CDX2, and TTF-1 was recommended to rule out a metastatic tumor. The whole-body scan demonstrated multiple osseous uptakes involving the skull, left rib, right sacroiliac (SI) joint and proximal femur. The prostate-specific antigen (PSA) was at the normal limit. IHC was negative for CK7, TTF-1, and Napsin A. CK20 showed a patchy reaction and CDX2 was diffusely positive (Figure 3).

Findings were more compatible with metastatic mucin-producing adenocarcinoma of gastrointestinal tract (GI) origin, especially the colon, and the patient was referred to gastric endoscopy and intestinal colonoscopy. No gastrointestinal (GI) problems were seen in these assessments. Thyroid, liver, and kidney function tests, blood tests, and chest radiography were also normal.

Complete workup including positron emission tomography (PET) scan was performed but the documents are not available. Based on the aforementioned data and oncologist consultation, the diagnosis of metastatic adenocarcinoma with unknown primary origin was made and he underwent chemotherapy based on a CUP syndrome protocol. Unfortunately, he passed away in May 2019.

## Discussion

Approximately 23% of oral metastases are the first indication of occult cancer elsewhere in the body [ 2 ]. Clinical signs and symptoms are variable and include bony swelling with tenderness, pain, ulcer, hemorrhage, paresthesia, pathological fracture, tooth mobility, trismus, and numb chin, but it may be asymptomatic [ 1 ]. A numbed chin is a major diagnostic indication of metastatic disease [ 7 - 8 ]. As a result, the possibility of metastasis should be raised in a patient who complains of paresthesia [ 7 ]. 

CUP comprises 2–3% of all epithelial cancers. The two main microscopic patterns include adenocarcinomas and undifferentiated carcinomas, although squamous cell carcinoma (SCC), neuroendocrine carcinoma, and uncommon histopathology comprise the remaining 10% [ 5 ]. The primary neoplasm may stay tiny and consequently escape clinical recognition or it may be vanished after seeding the metastasis or has been removed by the body’s defenses [ 6 ]. The most frequent primary locations recognized at autopsy include the lung, the pancreas, the GI (colon, stomach, bile duct, and liver), and the urogenital tract [ 5 ]. Metastases in lymph nodes, lungs, liver, or bone are the most common clinical manifestation of CUP. The majority of cases show disseminated metastasis. Clinical symptoms related to the involved organ and the extent of metastasis. In asymptomatic patients, it may be discovered incidentally through radiography [ 9 ]. IHC assessment helps determine the type of tumor [ 5 - 6 ]. The expression of cytokeratins (CKs) is very useful in determining the subtype. For instance, the profiles CDX2+, CK20+, and CK7- are typical of colon cancer, although the profiles CK7+, WT1+, PAX8+, and CK20- are characteristic of ovarian cancer [ 10 ]. CDX2 is the most well-known marker for the diagnosis of metastases originating from the GI. Moreover, the expression pattern is also significant. Colorectal malignancies demonstrate uniform positive stain; other site carcinomas show variable or focal staining [ 11 ]. In the present case, almost all neoplastic cells were positive for CDX2. Napsin A indicates pulmonary origin and co-expression with TTF1 is highly specific for pulmonary adenocarcinomas [ 11 ] that both of them were negative in current case. GATA3 is a sensitive marker for breast and urothelial carcinomas [ 11 ]. Asking about family history is important [ 6 ]. It has been stated that there is a familial cancer predisposition in CUP cases [ 10 ] and the metastatic site shows a familial clustering pattern. On the other hand, CUP patients’ relatives reveal an increased risk of CUP, malignancies of the lung, pancreas, or colon themselves [ 5 ]. Liver and kidney function tests, blood tests, chest radiography, mammography, or a PSA test should also be evaluated [ 6 ]. Bronchoscopy or colonoscopy should be done only when IHC findings or clinical features are highly indicative of lung or colon malignancies. TP53, K-RAS, and CDKN2A are most mutated genes [ 5 ]. An integrated PET-computed tomography (CT) is also needed to find the location of primary cancer [ 5 ]. Salem *et al*. [ 12 ] reported a 75-year-old woman with cervical lymphadenopathy that clinical and CT did not reveal any other site involvement. Lymph node biopsy showed metastatic SCC. F-18 FDG PET/CT imaging demonstrated a small lesion in uvula and biopsy showed SCC of uvula. 

In head and neck region, the most common occult primary site is the oropharynx and hypopharynx respectively [ 13 ]. On the other hand, cervical lymph nodes are the majority of CUP cases in this region [ 13 - 14 ]. SCC includes 53–77% of all head and neck CUPs [ 14 ]. The level of the metastasis can give evidences to predict the site of the primary lesion. In the case of SCC, metastasis to levels I, II, and/or III apparently shows that the primary origin is in the oral cavity or oropharynx. Moreover, in the case of adenocarcinoma, metastasis to level IV or V suggests that the primary neoplasm is located in the thyroid gland or thoraco-abdominal area [ 13 ]. In metastatic SCC cases, screening for association with human papillomavirus (HPV) and Epstein-Barr virus (EBV) is a beneficial diagnostic method to define the unknown primary location [ 15 ]. EBV positivity supports the nasopharyngeal origin. The evidence of HPV and P16 overexpression suggests the oropharyngeal origin. In some cases, the primary location has never been recognized. However, it maybe finds during follow up after initial treatment [ 13 ]. Oriyama *et al*. [ 13 ] described a 69-year-old female patient with metastatic cervical lymph node in which maxillary primary intraosseous carcinoma was found 6 months after the initial treatment. However, Aro *et al*. [ 14 ] mentioned that diagnosis of a primary later in the follow up does not affect the survival. 

Small proportions of CUP patients show a favorable prognosis, and attain meaningful improved survival. Nevertheless, most patients include the unfavorable subtype [ 16 ]. If there is a single metastasis, local radical surgery or radiotherapy is recommended [ 9 ]. However, more than 75% of CUP patients show multiple metastases, and systemic chemotherapy is recommended [ 9 ]. The majority of patients do not respond to chemotherapy and the medium overall survival rate is estimated about 6 to 10 months [ 11 ]. Conway *et al*. [ 16 ] stated that this one-size-fits-all attitude does not reflect the heterogeneity of these neoplasms, and underline the necessity for enhanced treatment stratification. Informed consent was obtained from the patient for publishing his radiography. 

## Conclusion

In conclusion, CUP of the oral cavity is an extremely rare event with poor prognosis. Numb-chin syndrome can be the first clinical sign of a disseminated metastasis. Oral and maxillofacial surgeons and pathologists should be familiar with this entity. 
